# The Influence of Host Stress on the Mechanism of Infection: Lost Microbiomes, Emergent Pathobiomes, and the Role of Interkingdom Signaling

**DOI:** 10.3389/fmicb.2017.00322

**Published:** 2017-03-02

**Authors:** John C. Alverdy, James N. Luo

**Affiliations:** ^1^Sarah and Harold Lincoln Thompson Professor of Surgery, Pritzker School of Medicine, The University of ChicagoChicago, IL, USA; ^2^Pritzker School of Medicine, The University of ChicagoChicago, IL, USA

**Keywords:** microbiome, injury, surgery, pathogens, sepsis, interkingdom interactions, interkingdom signaling

## Abstract

Mammals constantly face stressful situations, be it extended periods of starvation, sleep deprivation from fear of predation, changing environmental conditions, or loss of habitat. Today, mammals are increasingly exposed to xenobiotics such as pesticides, pollutants, and antibiotics. Crowding conditions such as those created for the purposes of meat production from animals or those imposed upon humans living in urban environments or during world travel create new levels of physiologic stress. As such, human progress has led to an unprecedented exposure of both animals and humans to accidental pathogens (i.e., those that have not co-evolved with their hosts). Strikingly missing in models of infection pathogenesis are the various elements of these conditions, in particular host physiologic stress. The compensatory factors released in the gut during host stress have profound and direct effects on the metabolism and virulence of the colonizing microbiota and the emerging pathobiota. Here, we address unanswered questions to highlight the relevance and importance of incorporating host stress to the field of microbial pathogenesis.

## Most Models of Infection Pathogenesis do not Incorporate Host Stress

It is well recognized, both in animal experiments and humans, that exposure to an infectious agent alone is insufficient to cause, consistently, clinical manifestations of the disease (Babrowski et al., 2013). Investigators often observe marked heterogeneity of disease manifestation when groups of otherwise similarly appearing and treated hosts are exposed to a given contagion (Connolly et al., 2015). It is for this reason that the “molecular Koch’s postulates” have been proposed to include changes in both host and pathogen phenotypes and their dynamic interaction when studying a single pathogen, or pathogen community, as a cause of an infectious disease (Falkow, 1988). It is well recognized that no two pathogens are alike, even they be from the same origin (Tenaillon et al., 2016). Microbes can also shift their phenotype *in vivo* in response to a variety of local environmental cues, each of which is particular to a given host, in a given tissue area, and in a given spatial context (Luong et al., 2014). It is for this reason that today, when experimentally modeling infection in small animals, we attempt to control as many variables as possible, such as breeding history, diet, housing conditions, etc. (Stappenbeck and Virgin, 2016). Nonetheless, unaccounted variability still exists frequently.

Traditionally, experiments are designed to detect “between-group” differences while manipulating genes in the infecting agent or host. Yet rarely are “within-group” differences of infection rates or mortality accounted for among the treatments so long as the between group differences are robust and statistically significant. What drives this heterogeneity of response within a highly homogenously treated group in a highly controlled environment? Here, we posit that the degree of physiologic stress of an individual subject plays a key, and regularly dismissed, role in the variability of infection-related outcome. To fully match all animals in a study, hourly measurements of numerous parameters (e.g., sleep, hunger, fear, anxiety, handling, etc.) would be necessary and integrated responses over the course of the experiment would need to be calculated. This is obviously not routinely performed and would be costly, if not impossible to achieve.

Yet in virtually every small animal experiment in which infection or mortality is used as the endpoint, there exists a high degree of variability in outcome that is rarely, if ever, reported or studied (Stough et al., 2016). What is often overlooked may be the emergent properties that develop in the infecting agent and the host as they interact with each other over the entire course of the host–pathogen relationship. Pathogen phenotypes are highly dynamic over the course of this interaction, as is the host physiologic response (hormones, cytokines, metabolome) before, during, and after the infectious inoculum is introduced (Shogan et al., 2014). A complex molecular dialog is developing as these two living organisms interact, exchange signals, and behave as one multi-cellular system (Rhee et al., 2009). Such dynamism will have a profound effect in shaping the social behavior of colonizing microbes.

**FIGURE 1:**
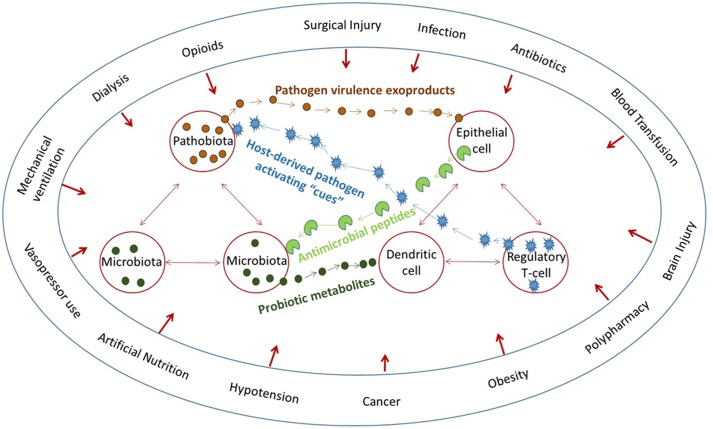
**The microbiome affects everything and everything affects the microbiome**. Multiple converging lines of bidirectional signaling between the host and microbiota and between the microbiota and pathobiota demonstrate that host circumstances directly affect both the microbiota and the immune system.

In order to model more precisely the host pathogen interaction, reductionist experiments with small animal models (i.e., *C. elegans*) and laboratory pathogens are used (Yuen and Ausubel, 2014). While much is to be gained from these reductionist models, they do not reflect some of the most challenging infections in humans, such as those that occur in modern intensive care units in the developing world (Rasigade et al., 2012). Patients, for example in an ICU are highly traumatized by procedural medicine, cared for under the most physiologically stressful conditions, and confined to the most hostile microbial environment (Zakharkina et al., 2017). Such patients are regularly exposed to healthcare associated pathogens that harbor unique antibiotic resistance patterns and highly virulent phenotypes (Busani et al., 2017). In addition, because of the promiscuous use of antibiotics to care for ICU patients, the protective action of the normal microbiota is essentially eliminated (Arrieta and Finlay, 2012). Hosts are vulnerable on two fronts, loss of the microbiome and the emergence of a virulent and resistant pathobiome (Krezalek et al., 2016). There is also evidence that physiologic or traumatic stress alone causes depletion of the host’s intestinal microbiome by unknown mechanism (Alverdy and Krezalek, 2017). Thus at the same time that compensatory host-derived signaling molecules are released during stress, which shift the phenotype of its colonizing flora, the normal microbiota are collapsing in abundance and function (Hayakawa et al., 2011). Such a scenario begs investigators to understand the role of physiologic and traumatic stress on infection-related outcome beyond their direct effects on the immune system and to apply a more holistic and systems biology approach to model infection, as it likely occurs *in vivo* (**Figure 1**).

## How Does Acute Host Stress Affect the Abundance and Function of the Microbiota?

It is now well established that following a sudden insult to the host, such as acute trauma, myocardial infarction, or burn injury, the intestinal microbiota decrease in abundance and function by greater than ninety percent (Shimizu et al., 2015). This observation may play an unappreciated role in the general consensus that a stressed host is more vulnerable to infection (Guyton and Alverdy, 2016). The scope and molecular details by which physiologic stress interacts with the intestinal microbiota and causes immunosuppression remains incompletely elucidated. However, ongoing investigations are beginning to shed some light on the mechanisms. In hospitalized patients who are critically ill, we often see a near complete ecological collapse of their endogenous microbiota, which is likely the result of both the patient’s active disease state and the selective pressure imposed upon them by modern intensive care efforts (Modi et al., 2014). Not only does the abundance of the microbiota become reduced in these patients, but low-diversity communities, often difficult to detect, tend to proliferate and are represented by highly resistant and virulent organisms such as *Candida albicans, Enterococcus* spp., *Staphylococcus* spp., and *Enterobacteriaceae* (Zaborin et al., 2014b). In one recent study by our group, Zaborin et al. (2014b), found that 30% of the critically ill patients had “ultra-low-diversity” microbial communities consisting of four or less bacterial taxa.

One of the most obvious and intuitive drivers of this ecologic collapse is the profound selection pressure imposed by the promiscuous use of antimicrobial agents. Extensive work has been reported to understand the effects of antibiotics on the microbiota (Modi et al., 2014). In 2010, more than 70 billion individual doses of antibiotics were consumed world-wide (Blaser, 2016). Broad-spectrum antibiotics can impact up to 30% of the bacteria among the human microbiota, resulting in severe loss of taxonomic and functional diversity (Francino, 2016). This dramatic shift in the microbiota can develop immediately following antibiotic administration, and can sometimes last for years after its cessation (Jakobsson et al., 2010). The perturbation of the endogenous flora has been linked to many disease states including obesity and autoimmunity (Francino, 2016).

While the effects of antibiotics are well studied and appreciated, the microbial collapse associated with critical illness is much more profound and broad when compared to exposure to antimicrobials alone. Many forms of host stress, independent of antimicrobial administration, have been shown to affect the composition and function of the microbiota (Mackenzie et al., 2017). For example, in patients undergoing gastrointestinal surgery, the use of opioid analgesics, withholding of enteral nutrition, and gastric acid suppression have all been shown to have profound effects on the microbiome (Krezalek and Alverdy, 2016; Levesque et al., 2016). Reuland et al. (2016) reported that the use antacids is associated with increased risk of extended-spectrum-β-lactamase producing *Enterobacteriaceae* carriage. Even surgical procedures themselves, such as colonic resection and reconnection, can be associated with a 500-fold increase in the abundance of *Enterococcus faecalis* (Shogan et al., 2014). This dynamic reality of microbiome stability further highlights the importance of understanding the complex host–microbiota interaction.

## How Does Host Stress Activate Pathogens to Cause Infection?

Attempts to elucidate the mechanistic details of this microbial shift have aimed mainly at the hypothesis that host stress causes immunosuppression (Vanzant et al., 2014). However, less well explored, is the possibility that host stress diminishes the protective intestinal microbiota, in both abundance and function, and that host stress signals activate colonizing “pathobiota” to express enhanced virulence (Lupp et al., 2007; Alverdy and Krezalek, 2017). It could be postulated that the intestinal microbiota “sense” that the host is under duress and decrease their growth rate and metabolism both anticipating that resources will be limited, and that the host cannot tolerate activation of its immune system by the intestinal microbiota (Babrowski et al., 2013). Alternatively, and in concert with this mechanism, could be the activation of intestinal antimicrobial peptides via IL-22, which is known to be elevated following traumatic and physiologic stress (Bingold et al., 2010; Rendon et al., 2013; Behnsen et al., 2014). In this way, the host keeps its intestinal microbiome “at bay” until which time recovery is established and homeostasis returns. The temporal dynamic of this response, the period of diminution, the refaunation process, and the species and community structure that are involved in this response remain to be clarified. Although some elucidation of this mechanism has been reported with the foodborne pathogen *Salmonella*; importantly, no host stress was imposed in the experimental model (Behnsen et al., 2014). Although such elegant and insightful models of *Salmonella* inform the mechanisms of its pathogenesis, they fall short in explaining why most humans exposed to the pathogen never develop an infection (Barak et al., 2009; Spencer et al., 2010). Several key questions remain unanswered. What are the mechanisms by which ingested isolates shift their phenotype to adapt to their new environment so they can express virulence factors that allow them to induce host cytokines (i.e., IL-22) that eliminate the microbiota? Do humans (and mice) who are stressed release host stress-derived compensatory factors that induce *Salmonella* to express these virulence factors that then determine if and how infection occurs (Krueger and Opp, 2016; Poroyko et al., 2016)? The last is a particularly important question given that we know that host stress depletes the microbiota, activates IL-22 (Bingold et al., 2010) (which further can deplete the microbiota), releases cytokines that directly signal bacteria to activate their quorum sensing circuits (Wu et al., 2005), and diminishes local resources (Long et al., 2008) (i.e., phosphate) in the local milieu (Long et al., 2008; Rendon et al., 2013).

Through a process recently termed “telesensing” (Roux et al., 2009), certain bacteria can not only sense their population density via quorum sensing, but also can detect and respond to host-stress derived signals such as opioids, cytokines, end-products of ischemia, immune cell environments, etc., that are unique to host tissues exposed to stressful conditions (Sansonetti, 2004). This type of interkingdom signaling has traditionally received little attention in the microbial pathogenesis field (Kendall and Sperandio, 2016). While certain physio-chemical cues, such as pH, redox state, phosphate, etc., are well known to influence bacterial virulence activation, an emerging area of interest is how host-stress derived compensatory “cues” drive colonization, invasion, virulence activation, and ultimately, the continuum of infection from symptom development to lethality (Mekalanos, 1992). We and others have described many of these host-stress compensatory elements, the receptors on bacteria to which they bind, and the downstream pathways that become activated leading to a shift in virulence (Seal et al., 2010). For example, the Gram-negative pathogen *Pseudomonas aeruginosa* can detect host physiologic disturbance by sensing opioids in the host environment, and in response, activate its quorum sensing virulence machinery (Zaborina et al., 2007). This process involves a complex and constant dialog between the pathogen and its host (Alverdy et al., 2000). The host secrete factors in response of microbial presence, the microbe in turn detects these signals and adjusts its virulence accordingly (Patel et al., 2007; Zaborin et al., 2014a). Many commonly encountered bacterial virulence mechanisms are subject to this additional level of host-derived signaling: biofilm formation, swarming, luminescence, toxin production, etc. (Palmer and Blackwell, 2008).

Host–microbe interkingdom signaling and telesensing are not novel developments. Because the microbiota and its human host co-evolved over tens of thousands of years, an elaborate signaling system exists between them (Sansonetti, 2004). It is well known that host catecholamines released during stress can induce bacterial growth, enhance colonization to host tissue, and virulence upregulation (Freestone and Lyte, 2008). In addition, the human “gut-brain axis” is an active area of investigation. We are just beginning to appreciate the level of involvement that the microbiota plays in the development of the human nervous system (Lyte, 2013). So far, we know that this gut-brain axis is a bidirectional dialog involving neural (e.g., GABA), endocrine (e.g., amines), immune, and humoral signals (Carabotti et al., 2015). In addition to host-produced signals, release and sequestration of inorganic compounds, such as phosphate, cooper, iron, have all been implicated in this host–microbe interkingdom signaling (Schaible and Kaufmann, 2004; Zaborin et al., 2014a). These complex mechanisms of communication help to maintain the mutualistic human-microbiome relationship, and is the product of millennia of co-evolution.

As such, the occurrence, course, and outcome of infection may be highly influenced by the degree of host stress, not only because stress has a direct effect on immune function, but because physiologic stress has a direct effect on bacterial behavior. In the context of human infection, rarely if ever, is host stress adequately instantiated into experimental models. Host genes are manipulated as are microbial genes, and pathogenicity described. However, a major flaw in this approach is the dismissal of the “within-group” variability in infection occurrence and outcome that may be the most informative of the host–pathogen dialog that must first occur for the process of infection to be initiated. As can be seen in **Figure 1**, converging lines of host–pathogen interactions make it extremely challenging to organize and study such a dynamic and fluid system in the context of a critically ill patient. It may be for this reason that no new therapies for sepsis in the critically ill have emerged in decades. Yet understanding how the microbiota collapse following host stress, how the pathobiota emerge to achieve a new state of equilibrium with the host, and whether the resilience of the host to achieve recovery depends on the ability of the microbiota to refaunate, remains a challenging but important line of inquiry (Sansonetti, 2004).

## Is Host Recovery from Stress Dependent on the Ability of the Microbiota to Refaunate?

**FIGURE 2:**
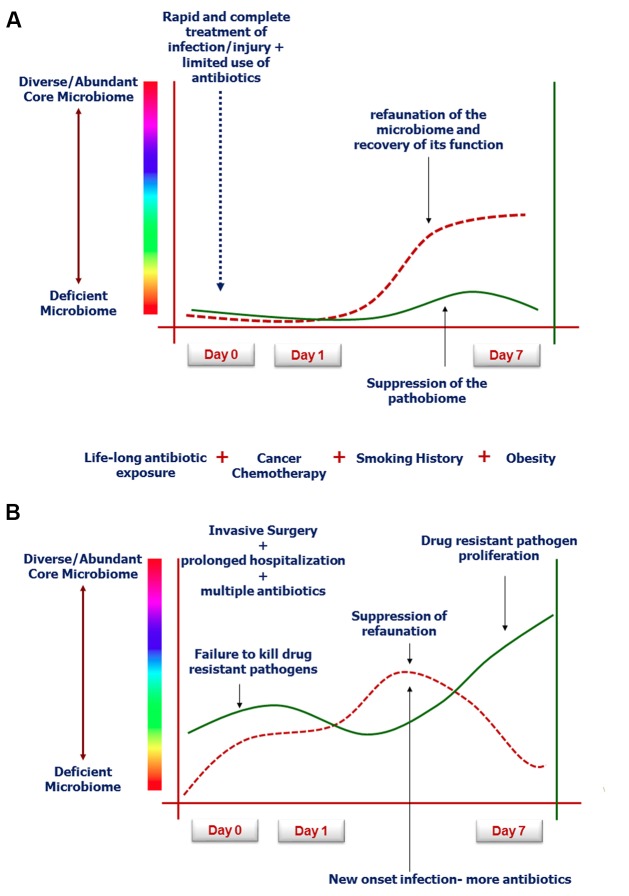
**Rate and degree of microbiota refaunation on recovery from severe catabolic stress such as following human critical illness (Guyton and Alverdy, 2016; Krezalek and Alverdy, 2016; Alverdy and Krezalek, 2017). (A)** Demonstrates typical response to successful modern medical care with limited antibiotic exposure and rapid resolution of the infection or injury (Modi et al., 2014). **(B)** Represents the aging patient population with multiple exposures to western diet, smoking etc, who have fragile microbiomes that cannot recover when invasive surgery and toxic agents are used to treat complex disorders such as cancer (Shogan et al., 2014; Shakhsheer et al., 2016).

While resilience to host injury and recovery from infection is generally attributed to a robust host immune clearance mechanism, emerging knowledge in microbiome science suggests that the intestinal microbiome plays a key role in driving a recovery-directed immune response (Shen et al., 2012). As described above, when a human is injured, both from the injury response itself and from its treatment by modern medicine, the intestinal microbiome can collapse in abundance and function (Shimizu et al., 2006). Yet as injuries are repaired and infections cleared with antibiotics, the ability of the host microbiome to refaunate is often considered to lag behind recovery, rather than to drive it (Jakobsson et al., 2010). However, equally plausible is the possibility that a previously healthy host (no smoking, limited previous antibiotic use, lean diet, regular exercise) may have a capacity to refaunate his/her microbiome to a greater degree than a previously unhealthy patient (Carlisle and Morowitz, 2011; Cosnes, 2016; Munck et al., 2016). The dynamics of refaunation and its correlation to recovery is poorly explored, however, with sequencing and metabolomics becoming more widely available and less costly, this can now be determined. Enhancing the refaunation process with fecal transplantation alongside therapies that are highly catabolic (bone marrow and solid organ transplantation) are underway and may further reinforce the plausibility of this concept (Kazerouni and Wein, 2017). The near disappearance of the intestinal microbiome following severe catabolic stress and injury, while adaptive prior to modern medicine, may be considered maladaptive in the present era, where highly toxic and invasive therapies (chemotherapy, radiation, severe burn injury) are needed to treat life-threatening diseases (Jenq et al., 2012; Pamer, 2016; Taur and Pamer, 2016). **Figure 2** depicts our theory involving the uncharted space in the intestinal tract that may play an unappreciated role in recovery from severe host stress.

## Conclusion

Pathogens bring their own unique life histories when they colonize or infected a new host. The complex dynamics of physiologic stress in the host drives these pathogens, and the microbial communities in which they co-exist, into a pathoadaptive process where genes are lost and found, and where new phenotypes emerge. Under such circumstances, emergent phenotypes among the colonizing pathobiota increase in frequency and compete for colonization sites and local resources. As stress becomes a persistent state and antibiotics are added to treat infections, microbial evolution speeds up as the emergent “pathobiome” enters an evolutionarily uncharted environment. As these pathobiomes compete for fixation niches, they become hidden from clinicians in protected sites where they do their dirty work at arms’ length from the immune system. Uncovering the dynamics of this host–pathogen interactome and the sites in which it occurs will lead to novel lines of inquiry and hypotheses to explain more completely the occurrence, course, and outcome of life-threatening infections that develop in the critically ill and all around the world.

## Author Contributions

JA: Conducted literature search and review; wrote, revised, and edited the manuscript. JL: Conducted literature search and review; revised and edited the manuscript.

## Conflict of Interest Statement

The authors declare that the research was conducted in the absence of any commercial or financial relationships that could be construed as a potential conflict of interest.
